# Supplementary material for: Inflammatory bowel disease and risk of idiopathic pulmonary fibrosis: A protocol for systematic review and meta-analysis
Source: PLoS One. 2022 Jun 24;17(6):e0270297. doi: 10.1371/journal.pone.0270297 (PMC9232144; doi:10.1371/journal.pone.0270297)
Supplement: S1 Table — (DOCX) [file pone.0270297.s002.docx]

| **S1 Table. Search strategy of PubMed.** | |
| --- | --- |
| Step | Strategy |
| #1 | “Inflammatory bowel disease”[MeSH Major Topic] |
| #2 | “idiopathic pulmonary fibrosis”[MeSH Major Topic] |
| #3 | (#1) AND (#2) |
